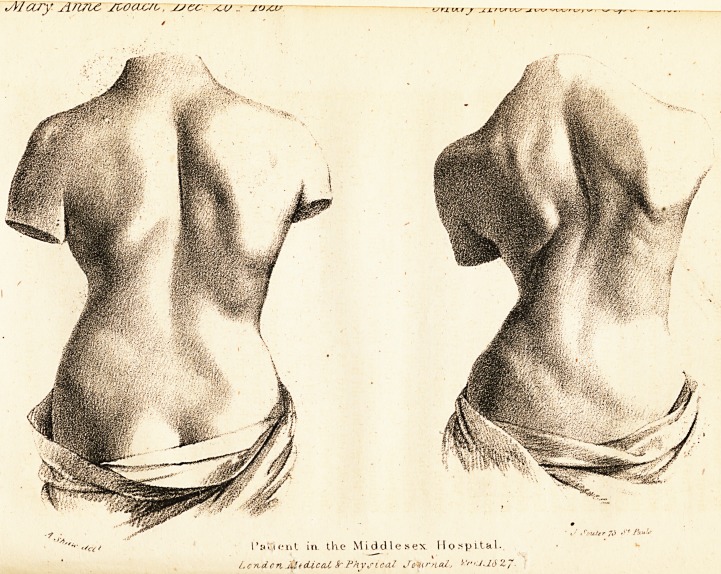# On the Different Modes of Treating Distortion of the Spine

**Published:** 1827-03

**Authors:** John Shaw

**Affiliations:** Surgeon to the Middlesex Hospital.


### DISTORTIONS OF THE SPINE.

### *On the different Modes of treating Distortion of the Spine.* By

John Shaw, Surgeon to the Middlesex Hospital.

In pursuing the subject of my former communication,* I shall endeavour to give a short account of the several theories which prevail on the causes of the *lateral* or *serpentine* twist of the spine, with descriptions of the different modes of practice. We shall thus be enabled to compare the plans recommended in England with those pursued in Germany and France.

The first question which naturally occurs is, whether the species of distortion represented in the Plate is as common among the poor as among the rich? I so entirely concur in the general opinion, as to assert that, for fifty young ladies who become twisted between the ages of eight and fourteen, there is not more than one *poor* girl similarly affected. It appears to be the children of the rich,—or, we should rather say, of the classes of society from the tradesman, whose daughters receive the advantages of a modern education, to the highest in the kingdom, that are especially subject to this peculiar twist of the spine, which may be remedied or prevented; while the poor suffer more from the almost incurable deformity which arises from rickets or a scrofulous disease of the vertebræ.

* See the Number for December, vol. lvi. 489–503.

As to the comparative frequency of deformity among boys and girls, I should say that, for one hundred young ladies who are twisted, there is not one young gentleman; while, among the poor, there are at least as many boys as girls deformed. Indeed, if we were to judge from the appearance of the people in the streets of London, we might believe that there are more crooked men than women in this class.

There is another distinction which, though important, does not seem to have been noticed: that the distortion of the spine in the poor generally commences in early childhood, while the twist is seldom observed among the rich before the children are sent to school, or begin their studies.

The question of how far this twist depends on bad health, or on a peculiarity of constitution, has been often the subject of discussion. Mothers are generally anxious to persuade us that their children, though twisted, are strong and healthy. There are certainly many girls who have slight curvature of the spine, in whom we can discover no signs of previous ill health, or of a weak constitution; but, in the greater proportion, we may observe one or more of the following circumstances:

Enlargement of the tonsils, or swellings of the glands of the neck; occasional discharge from the ears; fretted eyelids; the nostrils, cheeks, and lips, chopped or scurfy; the teeth bad, or small and irregular in form; the skin in different parts of the body affected with slight psoriasis, and not easily made to perspire, or so thin and transparent that the small veins are visible; the palms of the hands clammy and cold, or rough and arid; the fingers long and tapering; the nails badly formed; the hands and feet cold, and very subject to chilblains. All these symptoms are seldom presented in the same individual, but any two are sufficiently indicative of the system being torpid, especially if the girl is listless and unwilling to join in active games; or, while walking, lifts the feet heavily from the ground, and rests on the heels, instead of rising with a springy and elastic action from the toes.

Without due consideration, it might be alledged that this torpidity of the system causes the distortion. But, although a girl with such symptoms is in more danger than another of becoming crooked, the peculiarity of constitution indicated by them is seldom the sole cause: if it were so, the poor should be the most liable to be twisted, since the symptoms enumerated above are more common among them than among the rich, and are aggravated by the want of warm clothing, by bad food, and bad air; yet the children of the poor, after the age of ten, rarely become deformed, while the children of the rich, although they have enjoyed every comfort and attention that a kind parent can bestow, and have been even remarkable for the beauty of their form, become twisted about this time. It seems, therefore, just to conclude that, although young ladies whose figures are twisted may have a weak constitution, they would not have become crooked in other circumstances of life.

This is a difficult question;* but, the more it is considered, the more shall we be convinced that the present system of education, and especially the means generally resorted to for preventing or curing a deformity at its commencement, are such that a girl, even of the best constitution, is in danger of becoming crooked; while those in whom the symptoms already described are present seldom escape. How else can we account for the healthy girls of the poor never being distorted in the same manner, or for the weakly rarely becoming so, after they have attained a certain age. Indeed, I have scarcely ever seen the serpentine twist among the poorer classes of society, except where girls have been engaged many hours during the day in needle-work, or in occupations which rendered their muscles inactive: in several, the natural change in the constitution had not appeared until a late period,—not before the age of seventeen. Under the latter circumstances, distortion seems (in every class of society) to come on very suddenly, and to increase very rapidly.

The symptoms of the serpentine twist of the spine are generally observed by a parent in the following order:

1st. The frequent attempts of the child, when nine or ten years old, to prevent the dress falling off one shoulder.

2d. One shoulder appearing higher or larger than the other.

3d. One of the collar-bones, or one side of the breast-bone, or the breast itself, appearing fuller than the other.

4th. A thickness of one side, and a sinking-in of the other.

* When only one of three or four sisters who have been brought up on the same system becomes twisted, we must believe that she is of a weaker constitution than the others. I have generally been able to discover a difference in the constitution, or to trace the deformity to some peculiarity in the habits, or to a more than usual degree of weakness following measles or scarlet fever. When the distortion is observed at so early an age as from two to six, I suspect that it proceeds from a decided fault in the constitution, and is consequently almost irremediable; and, when it occurs in a boy, at any age, I believe it is also from this cause, unless he has been brought up under the same restraints as a girl.

5th. One hip appearing to project, or, as the mother expresses herself, "growing out."

6th. One leg appearing shorter, and the habit of standing on one leg, with a hand behind the back catching the opposite elbow.

7th. A peculiarity in the manner of walking; one foot being swung round, and one shoulder thrown forward. This habit of walking sideways is particularly observable when the girl is entering a room, or going up to a stranger.

When the girl reaches the age of twelve or thirteen, her figure is so evidently twisted, that the mother consults a surgeon, and he points out a curve of the spine from side to side, or rather of a serpentine form. Although this curve was probably never before observed, it has been gradually forming from the time the difficulty of keeping the clothes on one shoulder was first noticed.

The following is an outline of the view I have been led to form on the immediate cause of this yielding of the spine:

A girl of nine or ten years of age, although apparently in good health, is liable to some of the symptoms enumerated at page 212; or a strong and healthy girl is sent to school, where for a great part of the day she is cooped up in a long, narrow, and perhaps cold room, with a number of girls; sleeps in the same room with several others; is not so well fed as at home; and the only exercise she is allowed to take is a formal and weary walk. A child of the strongest constitution, under such restraints, soon becomes as liable to be crooked as those who are originally weak.

Children thus exposed to have their natural functions disturbed are liable to distortion, and from their generally appearing weakly and dejected previously to its being discovered that the spine is twisted, a lurking and insidious disease of the vertebræ is often supposed to be the cause of the apparent ill health, and also of the yielding of the column. But the most popular theory is, that the curvature is the consequence of sitting awry, of standing on one leg, of the habit of stooping, and of sitting carelessly while writing, drawing, or playing.

Such habits may assist in producing distortion, and will certainly increase it when once formed; but I suspect that the anxiety to prevent girls sitting negligently is one of the most fertile sources of distortion: I mean the frequent injunctions to a girl of ten or twelve years old, to "hold herself up." When we try to sit erect and stiff, as the little girl is ordered to do, only for a few minutes, we feel a weariness and pain at the loins, which we remove by stooping forwards or lying back. Were a girl to do so, it would be observed, and she would be reproved; but, as it is not in her power to keep herself in the desired posture, she must relieve herself in some way, and she generally does so by allowing the lower part of the spine to sink to one side, which is not so much observed as stooping or lying back. If the chair is so formed that it affords scarcely any support, or if she be placed on a music-stool or bench, it is cruel to blame her, because the position is that into which the column naturally falls if she is not allowed to stoop or lie back; as may be proved by placing a well-formed girl, of ten or twelve years old, on a music-stool. If the lower part of the spine be observed after she has attempted to sit erect for a quarter of an hour, or even for a few minutes, it will be found to sink to one side.

A girl gradually gets the habit of sitting in this way, until it is remarked that her shoulders are uneven, and that the clothes slip off one of them.* As the mother is not aware that the cause of the inequality in the height of the shoulders is the yielding of the base of the column, she begs the child to keep her shoulders straight, and perhaps puts on braces or a back-board to keep them even. In this way the bend at the lower part of the spine is overlooked, and, the ligaments gradually yielding, it increases to such an extent, that the child, to preserve its balance, makes a second curve in the spine: and now the mother is puzzled, for she finds that there is not so much inequality in the height of the shoulders, but that one appears larger than the other.

When this has taken place, the serpentine curve and the perpendicular twist are fully established; the ligaments, the intervertebral substances, the bones themselves, are all, to a certain degree, altered in form; and the muscles are not only daily becoming weaker, but more irregular in their actions, from the situation of their origins and insertions being changed. The girl is now in a critical condition. If the mother follows the advice which has been but too frequently given by men of eminence, to attend only to the general health,—or if she be induced to entrust her child to the care of a machinist or stay-maker, the distortion will get

* In the greater proportion of cases, the lower curve is to the left side, and the clothes at first slip off the right shoulder, (i.e. from the left shoulder being the highest;) but, when the second curve takes place to the right side, the right shoulder projects and gradually becomes higher, while the upper part of the left hip appears to "be out."

rapidly worse, and all the symptoms described at page 213 will appear in quick succession.*

Several distinct modes of treating this species of distortion prevail in England. One patient is rigidly confined for months to the same position; another performs certain violent exercises for years; a third is rubbed and shampooed; a fourth wears artificial supports, such as stays, collars, &c.; a fifth submits to attempts to replace bones alledged to be dislocated; a sixth is treated by leeches and blisters, or by caustic and moxa; and many are advised to trust merely to attention to the general health.

The variety in distortion is so great, that there are many where none of the systems of treatment hitherto proposed have been successful, and for which probably no remedy will be discovered. We know of no plan of treatment which will remedy a distortion where there is anchylosis of the vertebræ or of the ribs; nor that will make the shape perfect where the deformity depends on a congenital deficiency in the size of one side, (or on one that may be traced from the period of teething,) a defect as common as a small eye or a diminutive arm or leg; nor has any mode of treatment been divulged that will restore the spine to its proper form, where a curvature is the consequence of a collapse or diminution in the size of the area of one side, from disease of the lungs.

Although the plans mentioned above are directly opposite, both in principle and practice, so many testimonies are offered in their favour, that we are almost bound to believe they have been each attended with a certain degree of success. The only way we can reconcile such conflicting statements is by supposing that they who have reported the success have not been aware that there are many kinds of distortion, each depending on a different cause; or that the proposers of the several plans have deceived themselves by attaching importance to success in one or two instances, and in forgetting

* I know that I shall stand excused for this expression by my professional brethren, who have done me the honour to consult me on the cases of their own children: they have been but too well convinced of the danger of trusting to the advice of attending to the state of the bowels, and to give tonics, with fresh air, shower-bath, spunging, friction, &c. When the spine is once curved, it is as little likely to be remedied by such means, as a crooked tree is to be made to grow straight by merely manuring and watering it. But, notwithstanding this apparently obvious conclusion, nine in ten of the girls who are much distorted have gone on for years, in the hopes that such means will be sufficient to restore the figure. The general health should, in all the stages of distortion, be particularly attended to: attention to it may prevent distortion, or it may perhaps be the means of checking a slight degree; but, when the curves are fairly formed, I believe that it alone will not prevent the rapid increase of the deformity.

that there are so many distinct stages in the progress of a common case, that every mode may at particular times be applicable. To me it has always appeared, that a judicious combination of the modes reported to be useful was the most likely way of remedying a distorted spine. Thus, since the serpentine curvature generally originates in weakness of the muscles of the back, it is best remedied at its commencement by appropriate exercises, and attention to the general health. In the second stage, the muscles, ligaments, and intervertebral substance acquire a certain form, the good effects of exercises are not so apparent, but the figure may be improved by artificial supports. In the third stage, the vertebræ themselves become altered in shape: the remedies proposed for the second degree are still useful, but, that the bones may again *grow into a natural form,* the spine must be stretched, and kept so during the greater part of the day and night. When the ribs and sternum have become much displaced and mis-shapen, the distortion may be said to be in its fourth degree. All the means proposed for the preceding stages must still be rigidly persevered in, but a variety of contrivances for compressing and remodelling the ribs must also be adopted. The fifth or last stage is anchylosis. In this, any one, or all the plans of treatment combined, are little more than palliatives, or preventives of further increase.

But, as far as I can learn, this system of combining and applying the means according to the nature of the case, is not generally pursued. One particular mode is trusted to; and this, I believe, proceeds from those who propose the several plans having theories of the causes of distortion peculiar to themselves. I shall endeavour to make a short analysis of these theories, and of the plans of treatment generally pursued.

*Plan of Treating Distortions by Confinement to the Horizontal or Inclined Plane.*

The plan of *rigid confinement to one position* is founded on the idea that the muscles of the spine are debilitated, and act irregularly, in consequence of irritation or disease of the vertebræ, and that repose is necessary to subdue this irritation, and to restore the muscles to a natural condition.

I shall venture to assert that, in nineteen of twenty cases of lateral curvature, there is no disease of the vertebræ; that the pain in the back and shoulder, supposed to be indicative of disease, may be relieved sooner by other means than by rest; and that there is seldom or never that peculiar wasting or spasmodic affection of the muscles which are so frequently observed where the vertebræ or ligaments are diseased. In those cases where the muscles of the spine appear smaller than natural, it will be found that they either correspond with the condition of the other muscles of the body, or that the patient has been in the habit of wearing stiff stays.

The effects produced by confining a girl to the inclined plane are often misunderstood: she may at first be relieved from a weary pain, and at the end of three weeks or a month the spine may be found straighter; but if the same plan be pursued for some months, in the hope of improving the spine still more, or with the intention of preserving the improvement which has already taken place, all will go wrong: the patient will be reduced to such a state of weakness as to be scarcely able to walk or stand, and, when she attempts to sit up, will sink almost double, or into a state worse than she was when she first laid down; and there are few cases where the general health is not materially injured. But, notwithstanding these results, so much has been said by men of great eminence in favour of the theory on which this plan of practice is founded, that the confinement of girls with slight distortion of the spine, for months, and even years, is still often recommended.

The first few days a girl is laid on her back, although she is easy, the confinement is irksome; but she presently gets accustomed to the position, and almost enjoys it, and, if she be allowed to sit up, begs that she may again lie down. This is used as an argument to prove that, before the girl lay down, the spine was in a state of irritation; that it was relieved, and is getting better; but that if she rises the irritation will return, as is shown by her wish to lie down again: and this is alledged to be a clear proof that she herself is conscious of the good effects of the position. But I have known this argument used from month to month, and even from year to year. If there were disease of the bones or ligaments, the reasoning would be sound; but, when applied to cases of common distortion, the distinction between cause and effect seems to be overlooked.

*Performance of Violent Exercises, a popular mode of treating Distortions.*

This mode of treatment was founded on the supposition that distortion proceeds from the muscles of one side being so defective in strength as not to be able to counteract the effect of their more powerful antagonists. This opinion has arisen from the appearance of the muscles on the concave side of the curvature, when exposed by dissection, being supposed to be indicative of their having been strong and contractile, while those on the convex side seem to have been elongated and relaxed. From this it was inferred that the muscles of the concave side overpowered the others, and thus drew down the spine. This theory appears satisfactory, until it is recollected that the curvature to which young ladies are subject, is not a bend of the spine to one side, as the term *lateral* would imply, but is of a serpentine and twisted form.* But the great mistake is in supposing the contraction and shortening of the muscles to be the *cause*, when it is the *consequence*, of the distortion. The muscles, like the ligaments, accommodate themselves to the relative situation of their origins and insertions. When the sternum almost touches the pelvis, in cases where the lower dorsal vertebræ are wasted, the muscles of the abdomen are only a few inches long: would it be said that the deformity was in this instance caused by an irregular action of the abdominal muscles, prevailing over those of the back? Or when, by the same disease of the upper dorsal vertebræ, the chin approximates the chest, and the œsophagus is not more than four inches in length, from the mouth to the stomach, would it be argued that the muscular coat of the œsophagus had pulled down the head? (for its fibres are as strong as certain muscles of the spine, which are alledged to draw it to one side.) Or if, on dissection of an arm, where the humerus had been shortened by a badly united fracture, so as to diminish the distance between the origin and insertion of the biceps, this muscle were found, as in the case related by Mr. Hunter, six inches shorter than that of the other arm, would it be said that the biceps had produced the deformity? The condition of the muscles in serpentine curvature is analogous: those between the vertebræ, ribs, and scapulæ become gradually altered in length, according to the position into which these bones have fallen. One of the best proofs that the change in the form of the muscles is not from spasmodic contraction, is in the skin also becoming diminished in length, in proportion to the approximation of the bones.

However, it would be of little importance whether the

‡ The lateral curve of the spine, or bend to one side, without a perpendicular twist of the column, is not nearly so common as the serpentine twist. There was lately a good example in the Middlesex Hospital, in consequence of a rheumatic affection of the muscles of one side; and I am now attending a young gentleman in a similar condition, which proceeds from his inclining to one side to relieve the irritation produced by a slight inflammation of some of the ligaments of the spine. When the spine has yielded in consequence of disease of the hip, we generally find that the curve is merely to one side: there is an example of this at present in Northumberland Ward.

theory were correct or not, if the treatment founded on it were successful. It is alledged to be so; but, so far as I can judge, although properly regulated exercises are useful in every case of distortion where no inflammation is present, they are equal only to the cure of the slightest cases of lateral curvature. I may be mistaken in this, but I am positive that even a slight distortion will be more quickly remedied by combining various modes of stretching and supporting the ligaments and muscles, with exercises, than by exercises alone.

The immediate good effect of exercises in slight cases, the rapid increase of strength in those who are much distorted, and the consequent improvement in their form, deceive many into the hope that a confirmed curvature may be removed by perseverance. But the result of the cases where I have fairly tried the effects of exercise, and the state I have found the spines of those who had faithfully performed them for many months under proper superintendence, have convinced me that, where the ligaments and bones are altered in form, exercises are inadequate to the restoration of the shape. The statement made by Dr. Collin, of Paris, corroborates this opinion: of ten patients whom he examined, not one had been cured by a long continuance in the performance of the exercises at one time in vogue in Paris; such as drawing up weights, climbing ropes, &c.

I am, however, fully sensible of the value of exercises, and of such as operate more immediately on the muscles of the trunk: but I, in common with others, over-rated their effects at one time. I am now convinced that, in cases of confirmed curvature, the only good produced by exercises is counteracting the debilitating effects of certain other necessary modes of treatment. This question is considered at much length in the Supplement to my work on Distortions. I shall now only beg my reader to consider whether he has not often seen people who are strong and muscular, and who have for many years been constantly engaged in active and laborious exercise, as much or more deformed than weak and delicate girls? This surely is a convincing proof that exercises alone will not cure distortion.

Since writing the above, I have seen a young lady, who, for the preceding eighteen months, had every day, excepting Sundays and during the holidays, gone some distance to be shampooed, and climb a rope and ladder, and pull up a weight by a strap to be fixed round her head. Her friends stated that she had become much stronger, and her figure had been improved. This I can readily believe; but the condition of her spine, after attempts continued for eighteen months to make it straight, may be judged of from the plan.

A

B

A, is the upper part of the spine; B, the lower. The line is reduced from one made by taking an impression on blotting paper of the spinous processes dotted with ink.

In this case no artificial supports had been worn, nor had any means been taken to keep the upper part of the spine extended. I have no hesitation in saying that, had these been combined with the exercises, instead of merely trusting to the girl resting herself in the horizontal position after she reached home, the improvement in her form would have been much greater. The only additional remark I shall make is one elicited by a consideration of this case: that exercises, if not varied according to the place and nature of the curve, may increase rather than diminish the twist. By referring to the sketches of the figures climbing the rope and mounting the ladder, given in the Supplement to my work on Distortions, it will be seen that the curvature in the upper part of the spine cannot be improved by such exercises.

*Treatment of Distortions by Artificial Supports.*

The system of treating distortions of the spine by *artificial supports* is founded on the theory that the column is not sufficiently strong to sustain the weight of the chest, head, and shoulders. This theory is in part correct, but, certain important facts having been overlooked, the plan of practice founded on it has been so often injurious, that many surgeons have condemned every form of artificial support as useless or hurtful.

When instruments are constantly worn, they produce bad effects; but, if properly managed as auxiliaries, they assist in keeping the spine straight, and afford the means of enabling the patient to walk or sit for a certain time, without any danger of the spine falling back into the curved state which we are endeavouring to remove by other means. But it is not surprising that prejudice should have arisen against the tremendous engines generally employed for this purpose.

However, although the appearance of many of these machines is sufficient to alarm a mother, the assurances of their being the only means of cure have overcome their scruples, and delicate girls, regardless of pain, have submitted to wear them until many distressing consequences have ensued. The shape of the bones of the face is often altered, and the teeth displaced in an extraordinary manner; or the muscles of the cheeks are wasted by the pressure of the chin-straps, so as to increase the peculiar character of the countenance sometimes observed in deformed persons; and many are marked for life by the scars of ulcers under the chin.

But independently of those effects, which are surely as bad as the deformity proposed to be remedied, other consequences equally distressing ensue. By the pressure of the heavy complicated iron-work on the hips, back, and shoulders, many of the muscular fibres are absorbed; and the muscles by which the column should be supported gradually become so weak, from want of use, that the patient must either submit always to wear an artificial support, or, after enduring torture for years, to fall into a state worse than before using the collar.*

But, although I object to instruments clumsily contrived, or to such as are used without due attention to the functions of the ligaments and muscles, I am so satisfied of the necessity of artificial supports, that I recommend them, in certain forms, in almost every case of distortion.

The discrepancy of opinion on this question is a source of great difficulty to the friends of patients, and especially to

* The principal objection formerly made to such instruments was, that the pelvis might be distorted by them; but I trust it has been satisfactorily shown that there is no danger of this except in rickets. A case showing some curious effects on the muscles of the neck by a long perseverance in the use of one of these instruments, is given in the Supplement to my work on Distortion.

those from the country, who are "anxious to take all the advice London can afford." By one eminent surgeon they are told all supports are injurious; by another, that perseverance in the use of them will certainly cure this deformity. About eighteen months ago, a patient, whom I had recommended to wear supports when she walked or sat up, consulted an eminent surgeon in the east end of London: his advice was, to burn them, and trust to nature. From the answers received from patients, I am led to believe that this is the advice generally given: but one of the highest authorities in London lately recommended quite an opposite mode of treatment for a young lady, who had been also a patient of mine. However, notwithstanding the deference due to a senior of great merit, I would venture to alledge that his advice was too much in the opposite extreme to that of trusting to nature; for the effect of encasing a girl in a machine from morning to night must be to debilitate the muscles of the trunk, and thus to add to the original cause of the distortion. The machine recommended in this instance was that generally known by the name of the "invisible back," invented by Callam, the truss-maker, in Great Queen-street, who died lately. I have always objected to this instrument, because, although it may form a good temporary support, it is so neat and ingeniously contrived to conceal the deformity, that parents are apt to be deceived by it. When used with precaution, and only as the means of giving support for a certain number of hours, it is not very objectionable; but, as it has been injurious in all the cases that I have known it to be worn according to the directions of the maker, I have preferred a simpler form of support, and one which does not confine the shoulders, and consequently does not interfere with the growth and expansion of the chest. When a girl with a slight curvature of the spine arrives at such an age that she is not likely to grow, this instrument may be useful in concealing the deformity; but still the danger of the muscles of the spine being gradually debilitated should not be lost sight of, but guarded against by exercising them actively for at least half an hour morning and evening.

The question of the propriety of girls wearing stays has been often canvassed: it is not easily answered.* I have en-

* "A curious edict was passed by the Emperor Joseph the Second, to restrain the use and fashion of stays: in the preamble, it set forth that they impaired the health and growth of the fair sex. In all orphan houses, nunneries, and other places of public education, they were strictly forbidden; and young ladies of the tered at considerable length into the discussion in the Supplement to my work on Distortion, where I have endeavoured to show (although tight stays may be one of the causes of distortion,) that, when the spine has once yielded, there should rather be an addition than a diminution of the means of artificial support, until the natural powers, by which the spine is enabled to sustain the superincumbent weight, are restored. But, although stiff stays may for a time prevent a curvature from getting worse, and even for the first few weeks appear to improve the figure, they are not calculated to correct it; because they do not afford the means of gradually elevating the spine, but only of preventing it from sinking. It is hence often found that, if stays of the same size be worn for some months, the bones, cartilages, ligaments, muscles, even the skin, acquire a certain form and length: I therefore always recommend such supports as can be gradually elevated according to the change produced in the curve.

In a young lady, twelve years old, from the 25th November to the 3d of January, the curve was so much straightened that she measured two inches taller. In another, between seventeen and eighteen, where the curvature was much greater, the difference in three months was four inches. I had gradually, during that time, raised the supports four inches. The increase which also takes place in the breadth of the chest, makes stays still more objectionable.

*Attempt to cure Distortion by reducing Vertebræ alledged to be Dislocated.*

The proposal to cure distortions by replacing vertebræ alledged to be dislocated, is founded on so mistaken a notion of the structure and physiology of the spine, and of the functions of the parts connected with it, that it scarcely deserves a serious refutation. The merest tyro in anatomy knows that the effect of a sudden and violent change in the position of a vertebra must be more or less an injury of the spinal marrow. But, notwithstanding the demonstrable fallacy of the opinion that lateral distortion proceeds from dislocation, cases have been detailed in former Numbers of this Journal, where it is court still persisting in the fashion, were threatened with the loss of the "customary indulgencies and countenances" bestowed on their class. Thus the use of stays was made a sort of immorality. The College of Physicians was enjoined to draw up a Dissertation in support of the royal edict, which was distributed gratis. But what can a monarch do against fashion? the liberty of the corset was soon re-established in Austria in its full severity."

stated that the curvature has been cured by replacing certain vertebræ alledged to be dislocated, and that this was effected by main force of pulling and pushing. The author has even seriously related the nervous phenomena resulting from the change he produced in the position of the vertebræ. It is to be hoped that he will be always as easily satisfied; for, were he to succeed in his endeavours, his patient would assuredly be paralysed. Happily it is scarcely possible to alter the position of a vertebra without a degree of violence that is not likely to be used; and, therefore, notwithstanding the application of a *windlass* to pull the bones into their proper places! there is little danger of the spinal marrow being torn across or squeezed.

The description given by patients of the manner they have been pulled for the purpose of reducing the dislocated bones, is sufficiently alarming; but, in cases of simple lateral curvature, the *attempt at replacement* is not so injurious as the rigid confinement to the horizontal position for months, under the pretence of preventing the *replaced vertebræ* from slipping out of their places.

I have seen several patients who had submitted for a long time to this system of treatment: each of them had become miserably weak. One young lady had been confined for three years, until a short time before I saw her. On my first visit, I requested her to lie down on the sofa; she replied, "May I?" and, on asking why she was afraid to lie down, she said that she had not done so for three years without having one person to keep her head steady, and another to her feet, that the bones might not again be displaced. On assuring her that she need not be afraid, she, with much eagerness, asked if she might turn in bed without any risk of the bones slipping out? for she had been strictly cautioned against indulging even in this degree of motion.

This young lady had been reduced to such a state of weakness by this system of restraint, that her parents became alarmed for her life, and changed the plan of treatment so far as to encase her in a machine like a cuirass. While supported by this, she could walk; but, although I was told that she had become stronger, the muscles of her back, when I first saw her, were like shreds of ligaments, and the spine was so stiff and unyielding that I suspected anchylosis had taken place. I, however, proposed to try what could be done by combining gentle exercises of the muscles of the back with proper rest and support. By persevering in this plan, she became in a few months strong and muscular, and even gained nine pounds in weight. The restoration of her health afforded a satisfactory proof that the symptoms of consumption, under which she was supposed to be sinking, depended entirely on the confinement. I do not hesitate to say that this young lady, who had been rigidly confined at home for years, and even carried in a litter to the sea-side, and laid out on the beech, should not have been at any one time confined to the same position for twenty-four hours, nor prevented from taking daily and active exercise.

However, I occasionally hear from sources on which I can depend, that this system of reducing dislocated vertebræ has improved the curve; and although, in the eight or ten cases I have seen, the patients had not been benefited, I can believe that it has been of service. The process of stretching and pulling (although done with the view of reducing bones said to be dislocated,) when combined with rest on the plane, may make the spine much straighter, in eight or ten weeks. But if the patient, instead of being now brought into a state of great activity by exercise, is confined to the same position for months or years, for the purpose of consolidating the spine, and preventing the bones, which the operator alledges he has reduced, from again slipping out, bad consequences, and particularly great debility, must assuredly follow.

The most likely way of restoring a crooked spine to its natural form is to combine the several modes, and take advantage of what is good in each. This may, in a great measure, be effected by the moveable plane described in my last communication. It affords the means of stretching the ligaments and muscles in whatever manner or degree we choose, and of retaining the vertebræ and ribs in their improved relations to each other. It also admits of the patient resting in particular positions, and of performing such a variety of exercises, that the weakening effects of the stretching, compressing, &c. are completely obviated.

By the combination of appropriate exercises, rest, and support, the patient, if previously in bad health, becomes strong, and would, I am persuaded, continue in good health, even were she not to quit the plane for months. But I never put a patient to such a trial: I permit them to sit up for four hours, at least, out of the fourteen, (counting from seven in the morning until nine in the evening,) and to sleep on a comfortable hair mattress. It is of great consequence to prevent the spine from sinking while the patient is not on the plane, as the cure often depends on a proper growth of the intervertebral substance, and even of the bones themselves. To give support during

Mary Anne Roach, Dec. 20. 1826

Mary Anne Roach, [illegible]

A. Shaw del.

J. Souter 73 St. Pauls

Patient in the Middlesex Hospital.

London Medical & Physical Journal, [illegible] 1827.

walking, we may use the long crutches employed by the Germans and French; but a girdle round the pelvis, with light moveable supports attached to it, is more convenient.

The long crutches of the Germans and French are of great use when patients are treated in the manner described in my last communication: they not only afford the means of support to the lower part of the spine, but of exercise, and of so active a kind as in a great degree to obviate the ill effects of the continuance in the horizontal position. The crutches are so long that merely the tips of the toes touch the ground, but still the patients move with great velocity on them. The effect, indeed, is rather ludicrous; for the head and neck are so sunk between the shoulders, that the appearance of several girls running along very much resembles the pictures given of a flock of kangaroos. But the appearance would be of little consequence, were the position good; but it is not, for the cervical vertebræ are curved.

When the girl sits at the piano, writes, or draws, the chair crutch may be used instead of the support.

As the treatment consists principally in directing the form of the several parts, it must be continued for a period proportionate to the growth of bone: being thus necessarily very tedious, the surgeon has often difficulty in prevailing on the patient and her friends to persevere. If a girl is at school, and obliged to attend to the ordinary duties, or if she is indulged at home, little good can be done, and much time (very valuable to the patient) is lost. To show how much may be effected, even in four months, when the surgeon has the complete management and direction of the patient's occupations, I have had casts taken of the back of a girl, who was lately a patient in the Middlesex Hospital. In this instance the process of treatment was seen daily by my colleagues and by the pupils, and frequently by visitors. I was assisted in taking the casts by some of the pupils and one of the house surgeons; the first immediately on the patient's admission, the second on her being discharged from the hospital. They are deposited in Handel's Ward.

The case was very unfavourable, as the girl was of a short stinted form, and had menstruated for eighteen months previously to her entering into the hospital: however, in four months, she became nearly four inches taller, and the increase in her muscular power may be judged of by comparing the casts.

The variety in distortion is so extensive, and there are so many cases* in which little can be done, that I am induced to direct attention to the following questions, before a plan of treatment is decided on, or an opinion given as to the probable issue of a case.

*As to the time the distortion was first observed:*—Was it at an early age, or between eight and fourteen? Was it after one of the exanthematous disorders, as measles or scarlet fever? Did the change in the constitution take place early or late? Was it so late as the age of seventeen? Did the distortion seem to come on suddenly, and increase rapidly?

*With regard to the nature of the curve of the spine:*—Is it acute in some parts, or in a general waving line? Is the curvature between the shoulders to the right or left? Is the spine curved merely from side to side, or so twisted on its axis as to produce a prominence on one side of the spine and a sinking on the other? Is the distortion apparent above the first dorsal vertebra; or is the curve between the shoulders greater or less in proportion than that at the loins? Is any pain felt on pressure? What is the nature of this pain? Is there anchylosis of the vertebræ?

*With regard to the condition of the ribs:*—Is there an evident difference in the size of the two sides? Does this inequality disappear when the spine is elongated? Has the patient ever had a cough, attended with pain in the side that is diminished in size? Do the individual ribs appear mis-shapen? Does the sternum project; or is it flattened?

*In regard to the question of the state of the constitution:*—What was the condition of the health about the time of teething? Has the girl been of late listless, easily fatigued, and unwilling to take active exercise? Has the countenance that peculiar character which denotes deformity? Is the state of the skin or glands such as to mark a weak constitution? Are there any symptoms of infantile paralysis or blight? What was the condition of the wrists, knees, and ankles, during childhood? Are the limbs crooked or straight?

We should, moreover, attend to the general character and form of the patient; as in some instances the curve is rapidly improved, while in others the progress is exceedingly slow. The cases most difficult to manage, (excepting where a scrofulous action is distinctly going on,) are of two kinds. In the one the bones are very small, and the whole figure so diminutive as to be almost dwarfish: in such cases there is

* Many of these varieties are described and illustrated by plates in my work on Distortion.

probably a degree of rickets. The other is quite opposite, and unfortunately is not uncommon: the girl has a heavy leucophlegmatic appearance, is square built, often round-shouldered, and strongly formed, but is at the same time so slow in her movements, and so torpid, that it is scarcely possible to induce her to do any thing with life or animation. It might be expected that the lower part of the spine in these girls would be sufficiently strong to support the chest and head, and that consequently distortion would not take place; yet it is not unfrequent. In these cases the ligaments of the spine also seem so much firmer than usual, that we can scarcely produce any effect by stretching.

## Figures and Tables

**Figure f1:**
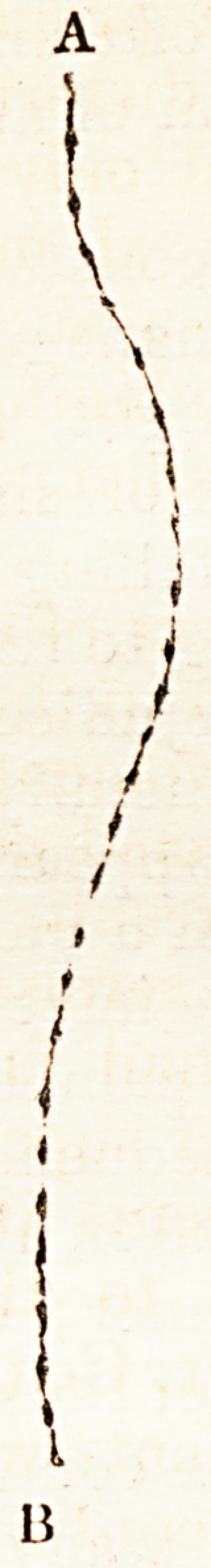


**Figure f2:**